# Assessing the role and effect of physician assistants in pediatric surgery: a prospective cross-sectional trial

**DOI:** 10.1097/JS9.0000000000003498

**Published:** 2025-09-24

**Authors:** Elena Johanna Weigl, Anna-Maria Hoebarth, Inge Eberl, Jan Goedeke, Oliver Muensterer

**Affiliations:** a Department of Pediatric Surgery, Dr. von Hauner Children’s Hospital, University Hospital, LMU Munich, Munich, Germany; b Department of Clinical Nursing Research and Quality Management, University Hospital, LMU Munich, Munich, Germany

**Keywords:** controversy, efficiency, nursing staff, physician assistants, workflow, workforce

## Abstract

**Background:**

Physicians spend an increasing amount of time on administrative activities. Physician assistants (PA) may help free-up time for nondelegable, patient-specific tasks. We began employing PAs in our pediatric surgery department in 2021 and evaluated their impact.

**Methods:**

We conducted a pre- and postinterventional study to measure the impact of adding PAs to our workforce. Parameters modelling workforce efficiency, including sign-out times, physician overtime, and discharge flow, were measured and compared over corresponding 5-month periods. Family, nursing staff, and physician satisfaction were assessed before (PA−) and after (PA+) hiring PAs, using Likert-scale questionnaires. All parametric quantitative data were compared statistically.

**Results:**

During the intervention period, sign-out to the on-call team occurred earlier (17:22 ± 0:47 hours PA+ vs. 18:21 ± 1:14 hours PA−, *P* < 0.001). Simultaneously, residents observed breaks more frequently (88% vs. 55%, *P* = 0.001), waiting time until discharge was reduced (1:38 ± 1:15 hours PA+ vs. 2:15 ± 1:44 hours PA−, *P* = 0.001), and effective discharge occurred earlier (13:08 ± 2:17 hours PA+ vs. 13:45 ± 2:26 hours PA−, *P* = 0.036). A total of 76 questionnaires by employees and 300 questionnaires by patients/families regarding satisfaction were analyzed. After the introduction of PAs, patient/family satisfaction improved from 1.5 to 1.3 (*P* = 0.041).

**Conclusion:**

The addition of PAs to our team had positive effects on patient and family satisfaction, quality of care, work efficacy, sign-out times, and patient discharge. These changes may translate into substantial economic healthcare savings, as well asimproved and more efficient patient care.

**HIGHLIGHTS**

- Quality of healthcare is impacted by shortages in healthcare professionals and the blurring of interprofessional responsibilities. Physician Assistants may help improve this shortfall, but are still not widely included or integrated into most European healthcare systems.
- Adding Physician Assistants to a pediatric surgical team increased patient satisfaction, improved physician workflow efficacy, decreased physician overtime, and led to earlier patient discharge.
- The described effects may translate into higher patient and staff satisfaction, improved efficacy of care, easier compliance with resident work hour restrictions and break policy adherence, as well as health expenditure savings. These findings should be evaluated further in prospective field trials.

## Introduction

Healthcare professional (HCP) shortages are common in many countries and may compromise the quality of care on a global scale. Although this problem is not new^[1]^, it is exacerbated by a growing number of administrative and nonmedical tasks. A survey conducted by the physician labor union in 2022 found that 57% of physicians spend more than three hours daily on organizational tasks, 66% of physicians felt HCP coverage was insufficient, and only 32% of physicians rated their working conditions as good or very good^[2]^. The profession of Physician Assistants (PAs) was introduced to potentially alleviate physicians in some technical tasks, as well as routine and administrative work^[1]^.

Originally employed in the military, PAs underwent surgical rotations to meet the needs of trauma care^[3,4]^. In some hospitals, PAs are employed on a routine basis, giving surgeons more time for non-delegable tasks while providing continuity of care^[4]^. PA education varies across countries. In our setting, PAs finish their 3-year academic curriculum with a bachelor's degree^[5]^.

A survey evaluating the role of PAs in pediatric surgery divisions in North America showed that almost half of the queried institutions incorporated PAs into their programs^[6,7]^. In the United States, PAs have not only become a vital part of pediatric surgery departments but are often advertised in the recruitment process of residency or fellowship programs as a means to enhance specialty training^[8]^. Despite some descriptive studies on the status quo and subjective impact of PAs in pediatric surgical practice^[3,6,7]^, the effects of PAs have not been systematically or prospectively investigated. In most European countries, the profession of PAs is just being implemented and defined^[9–12]^. In Germany, the first PA program was initiated in 2005, and at this time, PAs’ responsibilities, education, and remuneration lacked uniform regulation^[5,9]^. Demand for PAs is high, with 88% of PAs being successfully employed during the three months following graduation^[13]^.

To our knowledge, this is the first pediatric surgery department in our country to integrate PAs into a team. This gave us the opportunity to conduct a prospective, interventional study on the impact of PAs on work efficiency as well as patient and HCP satisfaction.

## Materials and methods

### Study design

The study compared two time intervals: before (September 5, 2021, “PA−”) and after (September 5, 2022, “PA +”) introducing PAs. The parameters assessed were specific workflow metrics, including discharge times, sign-out time to the on-call team, physician overtime, patient/family satisfaction, and HCP satisfaction. A training period of 6 months was allocated to the new PAs so that the postintroductory phase was not influenced by the learning curve. Data acquisition was performed for the same months of the year (May through September) to control for seasonal differences in workload. The study was approved by the local ethics board (reference number 21-0442). This study was registered with the LMU Research Registry and followed the (STROCSS) strengthening the reporting of cohort, cross-sectional and case-control studies in surgery guidelines^[14]^.

### Setting

Our pediatric surgery department is one of the largest academic tertiary referral centers for pediatric surgery in Germany. It performs more than 2000 operations per year. The team consists of 27 registered nurses on the pediatric surgery in-house ward and 20 physicians, of which 12 are postgraduate trainees and 8 are specialist pediatric surgeons. The pediatric surgery ward has 18 in-house beds. The treatment spectrum is broad and includes all pediatric surgical subspecialties. The physicians are responsible for the in-house ward, outpatient clinic, emergency department, intensive care units (pediatric and neonatal), staffing the operating room, and in-house consults for pediatric services. The pediatric surgery department has accredited pediatric surgery residency and fellowship programs.

### Intervention

On the November 1, 2021, two PAs were introduced to the pediatric surgical team. One had a total of 8 years of prior work experience in the ambulatory care sector, and one was a novice PA. The defined responsibilities of the PAs included admission and discharge of patients, documentation, scheduling of diagnostic/therapeutic procedures and consultations, assisting with rounds, and assisting with and performing independent bedside interventions (including dressing changes, cast care, drain removal, blood draws, and catheter placement). For training purposes, our novel PAs were doubled with residents for the first three months while adjusting to hospital-specific workflows and continuously taking on more supervised responsibility over the following three months. This training period was implemented before the study observation period to avoid a learning curve bias.

### Outcome parameters

The difference between expected and effective discharge times was defined as the primary outcome parameter. This parameter was selected to represent the efficiency and functionality of the discharge process.

Secondary outcome parameters were the starting time of bedside morning rounds, time of list-based afternoon rounds, resident sign-out to the on-call team, observance of legally required breaks, amount of overtime, and time dedicated specifically for resident training. Patient/caregiver and HCP satisfaction scores were also assessed.

### Data collection

Patient/family satisfaction was evaluated using the validated quality and satisfaction assessment tool of the Association of Pediatric Hospitals and Departments in Germany (*GKinD, Gesellschaft der Kinderkrankenhäuser und Kinderabteilungen in Deutschland e.V.*), which is similar to the Child Hospital Consumer Assessment of Healthcare Providers and Systems (HCAHPS)^[15]^. The results were calculated centrally and independently for yearly quarters. They are part of the routine standardized evaluation of pediatric hospitals throughout Germany. In the GKinD questionnaire, satisfaction was measured using a grading system, with 1 being the best achievable mark and 6 being the lowest. For this study, the results of the second and third quarters of 2021 and 2022, respectively, were used in accordance with the above-mentioned intervals.

Documentation of workflow efficacy occurred on weekdays, corresponding to the PAs’ scheduled work days. The workflow was documented using a chart of the time of admission, time of expected discharge, effective discharge, and completeness of discharge papers for each patient. The chart was completed by the patient manager to allow for consistent and independent documentation. Patients discharged on the weekend, on public holidays, or after PA and surgeon sign-out were excluded. The surgeons’ workflow was documented on weekdays by the residents responsible for the ward. HCP satisfaction was evaluated for physicians and nursing staff using a voluntary and anonymous monthly survey (Supplements 1 and 2).

### Statistical analysis

Data for workflow documentation and patient/family satisfaction were tested for normal distribution, and parametric tests were used. Means and standard deviations were calculated. For numeric variables, Student’s *t*-test was applied, and for nominal variables, the chi-square test (χ2) was used. Nonnormally distributed parameters were compared using nonparametric tests. The median and interquartile range (IQR) were calculated, and the sign test or Mann–Whitney U-test (U) was used, as appropriate. Data are presented as absolute numbers, followed by percentages. Statistical significance was set at *P* < 0.05. All statistical tests were two-tailed. Missing data were also reported. Statistical analysis was performed using IBM SPSS Statistics 26 (Statistical Package for the Social Sciences, version 26, IBM, Armonk, USA).

## Results

### Workflow efficacy

**Figure 1.**
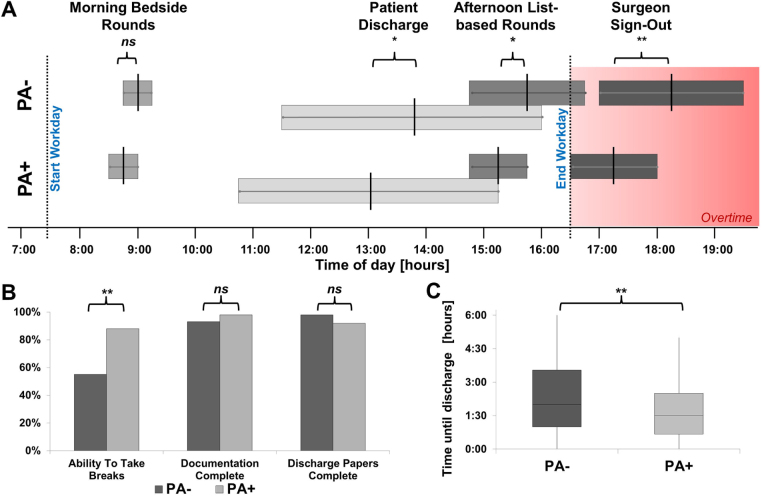
Graphical description of workflow and work efficacy in a pediatric surgery department before (PA-−) and after (PA+) integration of PAs into the team. Patient discharge, afternoon list-based rounds, and team sign-out occurred earlier after the introduction of Pas. (A) Required breaks were observed more frequently after PAs were introduced, while documentation remained equally complete. (B) Time until discharge (C) was significantly reduced. (*P < 0.5; **P < 0.01; ns, not significant).

A total of 85 days of physician-documented workflows were available for the analysis (44 days PA, 52%). The time of discharge was documented in 294 patients (184 PA−, 63%). Figure 1 shows a schematic diagram of the workflow that compares both intervals. There was no difference in the start time of the surgical morning rounds for both periods (8:55 ± 0:22 hours PA− vs. 8:49 ± 0:21 hours PA+, *P* = 0.20). Afternoon rounds occurred significantly earlier than before PA implementation (15:43 ± 0:51 hours PA− vs. 15:20 ± 0:37 hours PA+, *P* = 0.017). The effective time of discharge of in-house patients was significantly earlier (13:45 ± 2:26 hours PA− vs. 13:08 ± 2:17 hours PA+, *P* = 0.036), and waiting times for patients between communicated and effective discharge were significantly reduced after the introduction of PAs (2:15 ± 1:44 hours PA− vs. 1:38 ± 1:15 hours PA+, *P* = 0.001; Fig. 1C). A total of 1550 in-house patients were treated in 2021 (PA-), whereas 1734 patients were treated in 2022 (PA +, an increase of 12%).

### Overtime

Surgeons’ sign-out to the on-call team was significantly earlier in the day after inclusion of PAs in the surgical team (18:21 ± 1:14 hours PA− vs. 17:22 ± 0:47 hours PA +, *P* < 0.001, see Fig. 1A). On average, sign-out occurred 59 minutes earlier after the introduction of PAs. The official end of the workday at our hospital was 4:30 ᴘᴍ. Therefore, the introduction of PAs led to a decrease of more than half of effective overtime (1:51 hours PA− vs. 0:52. Additionally, compulsory breaks for surgeons required by national physician labor law were observed in 55% (24/44 days) of the days before PAs and in 88% of days with PAs (36/41 days, *P* = 0.001) (Fig. 1B).

Overtime and break time were not evaluated for other HCPs, as adherence to national labor laws was observed more strictly with less variability in these professions.

### Patient satisfaction and perceived quality of care

Patient/family satisfaction and quality of care questionnaires for 300 families were included in the analysis (101 PA−, 34%). Table 1 presents an overview of the items before (PA−) and after (PA+) the introduction of PAs. Patients and caregivers were generally very satisfied during both intervals, with scores ranging between 1 (very good) and 2 (good) for all items except for waiting times on admission. There was a significant improvement in perceived satisfaction in terms of the number of items highlighted in Table 1. After the introduction of PAs, overall patient satisfaction improved significantly from 1.51 to 1.30 (*P* = 0.041) during the intervention.

**Table 1:** Results of the GKinD patient satisfaction and quality of care surveys on the pediatric surgery ward before (PA-−) and after (PA+) the introduction of PAs. Scale from 1 to 6 with lower values being better

Item	PA−		PA+		Significance (*P*)
	*N*	Mean ± SD	*n*	Mean ± SD	
**The interview at admission was understandable and empathic.**	**83**	**1.35 ± 0.86**	**187**	**1.16 ± 0.62**	^a^**0.042**
HCP took their time to care for my child and me.	84	1.35 ± 0.81	173	1.29 ± 0.90	0.665
Waiting times at admission were appropriate.	83	2.04 ± 1.76	176	2.02 ± 1.46	0.949
The nurses were friendly towards my child and me.	92	1.10 ± 0.49	179	1.10 ± 0.45	0.964
I had trust in the nurses.	89	1.18 ± 0.51	165	1.18 ± 0.60	0.978
The physicians were friendly towards my child and me.	91	1.18 ± 0.63	178	1.11 ± 0.49	0.323
**I was well informed regarding necessary medical measures.**	**89**	**1.45 ± 0.88**	**179**	**1.25 ± 0.70**	^a^**0.041**
My questions to the physicians were answered comprehensible.	90	1.26 ± 0.74	178	1.13 ± 0.48	0.110
**I had trust in the physicians.**	**89**	**1.33 ± 0.93**	**175**	**1.11 ± 0.53**	^a^**0.016**
**The fears of my child were acknowledged and taken seriously.**	**89**	**1.21 ± 1.15**	**174**	**1.00 ± 0.83**	^a^**0.047**
**My own fears and I were acknowledged and taken seriously.**	**88**	**1.45 ± 1.23**	**171**	**1.13 ± 0.73**	^a^**0.009**
The pain of my child was acknowledged and taken seriously.	87	1.11 ± 0.64	176	1.09 ± 0.75	0.751
During medical procedures, the reduction of pain for my child was always aimed at (taking blood samples, punctions, change of wound dressings).	86	1.14 ± 0.78	173	1.02 ± 0.55	0.149
Our privacy was respected.	85	1.48 ± 1.19	175	1.46 ± 1.04	0.893
My child and I were always treated with respect and dignity.	88	1.16 ± 0.64	178	1.16 ± 0.64	0.983
**Information and preparation for my childs discharge was good.**	**86**	**1.62 ± 1.27**	**177**	**1.35 ± 0.78**	^a^**0.038**
**Preparation and guidance on how to care for my child at home was good.**	**84**	**1.37 ± 1.13**	**175**	**1.16 ± 0.52**	^a^**0.042**
I know whether I have to continue giving medications at home.	83	1.05 ± 0.87	181	1.03 ± 0.46	0.854
I know whether I have to see a paediatrician for follow-up.	84	1.15 ± 0.95	177	1.10 ± 0.60	0.585
**During this hospital stay I have felt good and safe.**	**90**	**1.51 ± 0.78**	**175**	**1.30 ± 0.78**	^a^**0.041**

PA−, before Physician Assistants; PA+, with Physician Assistants; SD, standard deviation.

Satisfaction is measured using the German school grading system with 1 being the best achievable, termed “very good” (≥90%) and 6 as the worst achievable mark termed “not sufficient” (≤40%). The Student’s *t*-test was used to calculate significance;

^a^ Significant difference (*P* < 0.05), Statistically significant differences are marked in bold and highlighted in gray.

### Healthcare professional (HCP)-survey

A total of 76 questionnaires were available for analysis (39 PA-, 51%). An overview of HCP-reported items is presented in Table 2. In both periods, HCPs reported that their patients were well cared for and satisfied. HCPs felt that they had sufficient time for their patients in 60% PA- and 50% PA+, *P* = 0.39. The nurses’ questionnaires (45 total, 27 PA−, 60%) showed a significant improvement of medical decision maker presence (surgeon or PA) in the surgical ward (20% PA− vs. 50% PA+, *P* = 0.006), and reported improved surgeon/PA reachability/availability (60% PA− vs. 80% PA +, *P* = 0.006). There was no difference in the time available for literature research, preparation for planned procedures, time for consultation, teaching sessions, scientific seminars, or simulation training.

**Table 2:** Healthcare professional survey. Items queried are listed in the left column

Item	PA−		PA+		Significance (*P*)
	*n*	Median (IQR) [%]	*n*	Median (IQR) [%]	
My patients were admitted on time.	34	100 (75–100)	32	80 (80–100)	0.485
My patients´ documents were complete.	38	75 (60–80)	36	75 (60–80)	0.678
**My patients had to wait for a room.**	**34**	**75 (45–100)**	**35**	**100 (75–100)**	^a^**0.021**
I have enough time for my patients.	39	60 (40–75)	37	50 (25–80)	0.390
I feel my patients are well taken care of.	36	75 (60–80)	34	80 (60–80)	0.926
I feel my patients are satisfied.	37	75 (60–80)	36	80 (60–80)	0.882
My patients did not have to wait for discharge.	38	50 (20–60)	36	45 (20–75)	0.658
After discharge, my patients were well informed.	37	75 (75–90)	35	80 (60–100)	0.291
I am content with the workflow of the pediatric surgical ward.	37	50 (35–60)	36	60 (40–60)	0.308
**Items in the surgeons` questionnaire:**					
I have time to prepare for my patients´ illnesses and interventions.	11	60 (50–100)	16	70 (60–80)	0.478
I have time to conduct literature research.	11	40 (0–80)	17	40 (20–70)	0.915
Attendance in assigned consultation hours.	10	100 (50–100)	13	100 (50–100)	0.938
Attendance at weekly teaching sessions.	11	75 (50–100)	15	100 (75–100)	0.564
Attendance at scientific seminars.	11	75 (0–100)	10	100 (45–100)	0.180
Time for pediatric surgical simulation training [minutes/month].	11	15 (10–45)	14	20 (11–60)	0.737
**Items in the nurses´ questionnaire:**					
**The surgeon/PA was always present on the ward.**	**27**	**20 (0–50)**	**18**	**50 (20–65)**	^a^**0.006**
**The surgeon/PA was always available.**	**27**	**60 (35–75)**	**18**	**80 (60–80)**	^a^**0.006**
I had trust in the surgeon/the PA.	15	80 (80–100)	34	80 (80–100)	0.934
**Items after PA introduction:**					
The introduction of PAs led to an improvement of the workflow on the ward.	–	–	35	80 (80–100)	*PA+ only*
The PAs were respected in their authority by the patients.	–	–	28	100 (80–100)	*PA+ only*
The introduction of PAs improved patient satisfaction.	–	–	27	100 (80–100)	*PA+ only*

PA−, before Physician Assistants; PA+, with Physician Assistants; IQR, interquartile range.

The Mann–Whitney U-test was used to calculate significance;

^a^ Significant difference (*P* < 0.05), Statistically significant results are marked in bold and highlighted in gray.

The introduction of PAs led to a subjective improvement in the workflow in the pediatric surgical ward (80%, IQR = 80%−100%). HCPs felt that PAs were respected in their authority by the patients (100%, IQR = 80%−100%), and that PAs led to an improvement in patient satisfaction (100%, IQR = 80%−100%).

In the open-comment section of the questionnaire, 15 employees stated that late patient discharge was the biggest problem in the workflow. After integration of the PAs into the team, the biggest point of critique was the perceived lack of clearly defined PA responsibilities. The PAs were described as the *“irreplaceable line of communication between surgeons and nurses on the ward.”*

## Discussion

This study systematically evaluated the potential benefits of incorporating PAs into an academic pediatric surgical department. To our knowledge, this type of interventional before-and-after comparison regarding the clinical impact of PAs has not yet been performed and therefore adds unique data on the benefits of incorporating PAs into surgical teams. In addition to increased patient and family satisfaction, we also demonstrated improved workflow in terms of earlier patient discharge, earlier sign-out to the on-call team, decreased physician overtime, and higher compliance with physician work breaks required by national labor laws. The positive impacts observed after the inclusion of PAs are highly relevant to the current discourse on healthcare reform and healthcare resource allocation in general.

Financial pressure in pediatric specialties is especially high in most healthcare systems because of limited remuneration for services^[16]^. This trend will most likely continue in the future with increased centralization and the closure of less profitable hospitals. The remaining centers will most likely struggle with providing enough HCPs to manage the necessary number of inpatient beds and ambulatory capabilities. To alleviate these effects, PAs have been proposed and implemented in more than 50 countries^[3,4,17]^.

The main hindrances to the broader employment of PAs in our environment are the lack of a uniform regulation of responsibilities, along with a deficit of knowledge and a lack of acceptance of the profession^[18–20]^. Our survey showed that legal questions on responsibilities were a concern for many HCPs in our team, despite intramural educational efforts. The World Health Organization (WHO) has identified broad country-specific differences in PA training, requirements, accreditation, and responsibilities^[17]^. In general, PAs are defined as “paramedical practitioners [who] provide advisory, diagnostic, curative, and preventive medical services that are more limited in scope and complexity than those carried out by medical doctors. They may work autonomously, or with limited supervision of medical doctors, and perform advanced clinical procedures for treating and preventing diseases, injuries, and other physical or mental impairments, common to specific communities”^[17]^. In the United States, PAs are allowed to independently run outpatient clinics with similar outcomes^[3,21]^. Furthermore, a cross-sectional survey revealed that 54% of PAs took calls, 44% applied casts and splints, 61% treated patients without supervision, and 85% assisted in surgery^[3]^. In the Netherlands, PAs are authorized to perform medical procedures and prescribe medications without direct supervision^[22]^. In Germany, PAs are not yet allowed to work independently. Nondelegable tasks of physicians include obtaining written consent, making diagnoses, performing interventions, prescribing medications, and performing invasive procedures^[9]^. Despite these restrictions, the inclusion of PAs in our team was associated with an increase in workflow efficacy and earlier surgeon signout.

General patient satisfaction and perceived quality of administered care, as metrics for good healthcare delivery, improved significantly after the introduction of PAs. The increase of 0.2 points may appear modest; however, the vast majority of average scores of participating hospitals range from 1.0 to 2.0, meaning that a 0.2 difference can be considered substantial. While this is the first prospective evaluation of patient satisfaction regarding the introduction of PAs, there are published international retrospective data showing high patient satisfaction regarding their experience with PAs^[23]^. Patient acceptance and awareness of the profession of PAs were good and increased with consistent practice^[23]^. In our evaluation, we found that respect for PAs and integration into our team, as well as acceptance of PAs from patients and families, were not an issue.

The quality of medical care should be considered when aiming for healthcare improvement. Numerous publications have shown that the introduction of PAs can lead to a reduction in complications and rehospitalization rates, as well as shorter lengths of stay and a decrease in treatment costs^[21,24–27]^. In our study, we evaluated these factors indirectly using satisfaction questionnaires. However, this is only an indirect approach; patients/caregivers felt significantly better informed and prepared for medical procedures, admission, and discharge. More importantly, they felt that their pain and fear were acknowledged more readily.

We also detected an improvement in continuity of care along with decision-maker availability. This effect is crucial for high-quality care, not only on a daily basis when surgeons are absent in the operating room or the emergency department, but also for long-term patient care. Patient data are lost over weeks and months during handovers because of surgeons’ on-call shifts, training sessions, and rotations to other specialties or hospitals^[28]^. While there is a plethora of physician extenders and advanced practice providers (APPs) available, including scribes, nurse practitioners, and coding assistants to redistribute specific tasks, PAs are trained as independent, multipurpose extenders, particularly for surgeons^[3]^.

Achieving decision-maker availability addresses another important factor of healthcare system reform: workflow efficacy. The main nondelegable work of surgeons and residents is performing operative interventions and/or committing to surgical training. When work on a surgical ward has the tendency to stall, a team member who can make autonomous decisions is essential to keep the system running^[28]^. After the introduction of PAs, we noted earlier afternoon rounds, earlier patient discharge, and the subsequent earlier availability of desperately needed beds. Although partially attributed to external factors, such as the subsiding SARS-CoV-2 pandemic, we observed an increase in patient numbers by 12% after PA introduction. Obviously, in this type of observational trial, it is impossible to precisely determine the cause and effect of the patient volume increase. However, a similar PA-associated increase in patient numbers has been reported for a level 1 trauma center^[29]^.

In our study, the addition of PAs led to a reduction in physician overtime, from 1:51 hours to 52 minutes daily. This may translate into substantial cost savings for the hospital. Cost-effectiveness is a complex calculation with many variables and outcome parameters, but considering that an hour of physician time was saved on each working day of the month (20 days), and that an hour of overtime costs the hospital at least 100 euros, the economic savings per physician would amount to around 2000 euros per month. Since the ward team consists of two to three physicians on a given day, total overtime saved would be at least 60 000 Euros in overtime, approximately the cost of two PAs in our healthcare system. We anticipate an increase in cost-effectiveness with further acceptance and standardization of the PA workflow in the future.

In addition, breaks required by German labor law were taken more frequently, increasing work-rule compliance and decreasing the legal risk of being sued for breaking labor laws. Despite these improvements in working conditions, subjective physician satisfaction did not improve, most likely because of the high, stable rates of job satisfaction in general. Adding only two PAs to a physician workforce of approximately eight consultants and 12 trainees may not be sufficient to create a more widespread impact due to the reduction in physician overtime. A more robust PA workforce may be necessary to produce further effects on the training curriculum. Interestingly, this phenomenon of *de facto* reduction in resident overtime and a contradictory subjective belief by residents that PAs had no impact on their working hours has previously been published for a surgical department in 2003^[30]^ and may be due to the unconscious fear of being replaced^[19,31]^. Future studies with longer study periods may be necessary to fully appreciate the effect of PAs on team dynamics and training.

Initially, an increased demand for HCPs was met with an increase in postgraduate trainees, as residents are responsible for providing care as part of their training^[32,33]^. However, employing more residents is expensive and decreases the quality of training as individual caseloads are diluted.

Specialist training and education usually come second to patient care in an economically strained healthcare system, in which residents carry most of the burden of administrative tasks, working hours are long, and workload density is high^[9,28,30]^. Residents are responsible for providing medical and surgical care as part of their training^[32]^, but independent studies and didactic teaching are hampered by long working hours, overtime, and high workload density^[28,30]^. In our study, overtime was reduced by integrating the PAs into our team. This has also been described for other university hospitals, in which PAs increase patient coverage and ensure appropriate residency training^[33]^. A large survey on the role of advanced care practitioners in pediatric surgery in North America described an enhancement of resident/fellow training in 85% of responses^[6]^. Achieving high-quality medical training means ensuring the conveyance of good patient care and leaving room for daily hands-on practice in a productive learning environment^[28]^.

Our subjective physician survey revealed no increase in attendance of lectures, seminars, or simulation training before and after the integration of PAs into our team, possibly due to ceiling effects or resistance to role changes.

In the future, PAs might participate in resident training, and a study investigating the ability of PAs to teach advanced trauma life support (ATLS) showed that PAs were comfortable teaching their own skills and leading interactive discussions with appropriate experience^[34]^. In an interview study, recommendations for the effective integration of PAs into residency programs were identified. These included a broader acceptance of the profession among residents, by assuring that residents do not become completely dependent on PAs, by avoiding that practical training of the residents is not compromised by PAs (e.g., that PAs are not substituted for residents in operative cases), and that the residents remain primarily responsible for their patients^[31]^.

In the United Kingdom, the implementation of physician assistants in the healthcare workforce, starting in the early 2000s, has recently faced controversy and backlash. In essence, physician shortages over time led to PAs taking over more tasks and decision-making. Some argue that some PAs are overstepping or are expected to overstep their competencies, including the execution of complex procedures, potentially endangering patients^[35]^. In fact, some high-profile deaths were reported in the lay press after receiving treatment solely by PAs, including the death of a 30-year-old actress from a pulmonary embolus. This has led the government and the National Health Service to review the role of PA in England. There is also a concern that PAs are more likely to be deployed in deprived areas, leading to inequalities in patient care and outcomes within the system. Recently, artificial intelligence (AI) has been increasingly explored in the surgical field to improve and standardize patient care^[36]^. Advances in AI in combination with the implementation of PA programs and resident training might allow for a more egalitarian, standardized, and safer healthcare administration. Moreover, a recent systematic review found that the role of PAs is not universally understood by patients and the public, although many patients were willing to be seen by PAs and viewed them as useful adjuncts to patient care^[37]^. Recently, some have called for argumentative discourse to redefine the role and competencies of PAs in the United Kingdom^[38,39]^.

Although we have not experienced this type of backlash from our physician or nursing staff, free-text responses within our survey indicated that most healthcare professionals in our department found it important that delegable tasks, competencies, and procedures needed to be clearly defined beforehand.

This study has some limitations. Owing to the single-center nature of our study, some findings may have been affected by locoregional factors. These results may not be generalizable to other hospitals with different workflows or staffing models. The prospective, interventional study design also precludes blinding and randomization. The before-and-after design is susceptible to temporal biases. Because patient and HCP satisfaction are multivariate and multilayered, attribution to a single factor is difficult. Nursing staff turnover, resident schedules, and other factors may also have had an effect. Nevertheless, this is the first and only systematic evaluation of the effect of PAs on a pediatric surgery department in Germany, and seasonal differences were minimized by choosing the same months for pre- and post-intervention observations. Finally, since the postinterventional part of the study was performed within 1 year of implementing PAs, some of the collected data may be affected by the learning curve of the system accepting the new workflow, including PAs. As PAs become more widespread and part of the standard of care, controlled interventional studies will become increasingly difficult to perform.

It has now been 4 years since hiring the first PAs in our department. Since that time, PAs have been completely integrated into our team. We have not detected any remorse or lasting conflicts among the PAs, nursing staff, and physicians. The duties and responsibilities have been transparently listed in a dedicated “PA Manual,” which is available online to all staff members. The patient volume increased further by approximately 20% without a corresponding increase in physician numbers or overtime. Patient satisfaction remained stable at a very high level compared with that of other national children’s hospitals. In hindsight, we are convinced that adding PAs to our workforce has sustained positive effects.

## Conclusions

Auxiliary professionals in the form of PAs are helpful in complementing nurses and physicians and thereby ensuring high-quality care in times of personnel shortages and fluctuations. The integration of PAs can help overcome these challenges and is associated with increased workflow efficacy and patient satisfaction. Clearly defined tasks, functions, and limitations are essential to avoid conflicts and make the best use of PAs in the healthcare environment.

## Data Availability

The anonymous raw data of this study are available upon reasonable request to the corresponding author.

## Research registration unique identifying number (UIN)

LMU research registry (21-0442).
